# Maternal Preconception Glucose Homeostasis and Insulin Resistance Are Associated with Singleton and Twin Birthweight of Neonates Conceived by PCOS Women Undergoing IVF/ICSI Cycles

**DOI:** 10.3390/jcm12113863

**Published:** 2023-06-05

**Authors:** Huahua Jiang, Yaxin Guo, Lixue Chen, Huifeng Shi, Ning Huang, Hongbin Chi, Rui Yang, Xiaoyu Long, Jie Qiao

**Affiliations:** 1 Center for Reproductive Medicine, Department of Obstetrics and Gynecology, Peking University Third Hospital, Beijing 100191, China; 2116395010@bjmu.edu.cn (H.J.);; 2 National Clinical Research Center for Obstetrics and Gynecology, Peking University Third Hospital, Beijing 100191, China; 3 Key Laboratory of Assisted Reproduction, Peking University, Ministry of Education, Beijing 100191, China; 4 Beijing Key Laboratory of Reproductive Endocrinology and Assisted Reproductive Technology, Beijing 100191, China; 5 Reproductive Medicine Center, Tongji Hospital, Tongji Medicine College, Huazhong University of Science and Technology, Wuhan 430074, China; 6 Obstetrical Department, Department of Obstetrics and Gynecology, Peking University Third Hospital, Beijing 100191, China; 7 National Center for Healthcare Quality Management in Obstetrics, Beijing 100191, China; 8 Beijing Advanced Innovation Center for Genomics, Peking University, Beijing 100191, China; 9 Peking-Tsinghua Center for Life Sciences, Peking University, Beijing 100191, China

**Keywords:** PCOS, preconception, birthweight, glucose metabolism, in vitro fertilization

## Abstract

Polycystic ovary syndrome (PCOS) can induce fertility and metabolism disorders, which may increase the prevalence of glucose metabolism disorders and cause health hazards to women and their offspring. We aim to evaluate the effect of maternal preconception glucose metabolism on neonatal birthweight in PCOS women undergoing IVF/ICSI cycles. We retrospectively analyzed 269 PCOS women who delivered 190 singletons and 79 twins via IVF/ICSI at a reproductive center. The effects of maternal preconception glucose metabolism indicators on singleton and twin birthweight were evaluated using generalized linear models and generalized estimate equations, respectively. The potential nonlinear associations were evaluated using generalized additive models. The analyses were further stratified by maternal preconception BMI and delivery mode to evaluate the possible interaction effects. Among PCOS women, maternal preconception fasting plasma glucose (FPG) and glycohemoglobin (HbA1c) had significant negative associations with singleton birthweight (all *p* for trends = 0.04). We also found an overweight-specific association between elevated maternal preconception 2 h plasma insulin (2hPI) and twin birthweight (*p* for interactions = 0.05) and a caesarean-specific association between maternal preconception HbA1c and singleton birthweight (*p* for interactions = 0.02) in PCOS women. Maternal preconception glucose metabolism may affect neonatal birthweight, suggesting the importance of preconception glucose and insulin management for PCOS women. Further large prospective cohorts and animal studies are needed to confirm these findings and investigate the potential mechanisms.

## 1. Introduction

Over the past few decades, the prevalence of impaired glucose tolerance (IGT) and diabetes mellitus (DM) has increased rapidly worldwide [1,2]. The cases of IGT and DM in adults aged beyond 18 years were estimated to be 374 and 451 million in 2017 and are predicted to rise to 587 and 629 million in 2045, respectively [2].

IGT and DM have become major health burdens affecting women of reproductive age and have caused increased health hazards to women and their offspring [3,4]. With the proposal of the developmental origins of health and disease (DOHaD) hypothesis [5], the effects of maternal glucose metabolism disorders (i.e., insulin resistance (IR), IGT, DM) during the preconception and pregnancy period on pregnancy outcomes and offspring have been widely valued [4,6,7,8,9]. However, globally, almost half of the women with DM (49.7%) were undiagnosed [2]. Moreover, IR, IGT, and DM often occur in polycystic ovary syndrome (PCOS), which affects 5–18% of women of reproductive age with lifelong high risks of reproductive, endocrine, metabolic, and psychological complications [10]. Mendelian randomization (MR) analyses have proven that fasting insulin and IR are the cause of PCOS [11]. In addition, PCOS women with insulin sensitivity status have a higher risk of gestational DM [12].

Studies have elucidated the adverse effect of abnormal glucose metabolism on pregnancy and neonatal outcomes in pregnant women. Hyperglycemic pregnant women were proven to have a high prevalence of caesarean section, preterm delivery, low one-minute Apgar score, respiratory distress syndrome, neonatal jaundice, admission to the neonatal ICU, infants born large for gestational age (LGA), macrosomia, and offspring who were overweight obesity obese at preschool age [9,13,14,15,16]. Rare mutations of GCK (i.e., rs1799884), a key determinant of fasting glucose, can cause fasting hyperglycemia and alter birthweight [17,18]. Maternal glycohemoglobin (HbA1c) and IR during pregnancy were also illuminated as altering neonatal birthweight [19,20]. Compared with the pregnancy period, the preconception period is essential for the growth and maturation of oocytes. An abnormal maternal environment may adversely affect oogenesis and subsequent offspring outcomes [8,21]. However, the available human data on the relationship between abnormal preconception glucose metabolism and neonatal outcomes are rather limited [4,7]. Moreover, although PCOS women are prone to glucose metabolism disorders, scarce studies have focused on the neonatal outcomes of PCOS women undergoing in vitro fertilization (IVF) or intracytoplasmic sperm injection (ICSI) cycles.

This study aims to illustrate the effects of maternal abnormal preconception glucose metabolism (i.e., abnormal glucose homeostasis, IR) on the birthweight of singleton and twin neonates conceived by PCOS women undergoing IVF/ICSI cycles.

## 2. Material and Methods

### 2.1. Ethics Statement

Approval was obtained from the Institutional Review Board of Peking University Third Hospital (no. 2021SZ—011), and all participants in this study signed consent forms.

### 2.2. Study Population

We retrospectively analyzed PCOS patients aged between 20 and 45 years old who underwent their first fresh IVF/ICSI cycles with autologous oocytes at the Reproductive Center of Peking University Third Hospital from 1 January 2018 to 30 December 2020. The diagnosis of PCOS was based on the Rotterdam criteria by at least two of the following three clinical features: oligo- or anovulation, hyperandrogenism (clinical or biochemical), and/or polycystic ovary [22]. The exclusion criteria were as follows: (1) history of ovarian diseases or iatrogenic ovarian injury; (2) uterine abnormality; (3) history of endocrine disease, autoimmune disease, or recurrent spontaneous abortion; (4) maternal or paternal chromosomal abnormalities; (5) undergoing in vitro maturation or preimplantation genetic testing cycles. Finally, 269 PCOS women who gave birth to a total of 190 living singletons and 79 living twins were included in the current study. Data on the baseline reproductive characteristics, cycle characteristics, and birth outcomes (i.e., neonatal birthweight, delivery mode, gestational age, and neonatal sex) were extracted from the internal database. The gestational age was computed according to the American College of Obstetricians and Gynecologists (ACOG) [23]. 

### 2.3. IVF Procedures

All of the enrolled patients received a standardized individualized controlled ovarian stimulation protocol according to their BMI and ovarian reserve characteristics (i.e., female age, antral follicle count (AFC), basal follicle-stimulating hormone (FSH)), as previously described [24]. Final oocyte maturation was triggered with 5000 to 10,000 IU hCG (Livzon) when at least two leading follicles reached beyond 18 mm. Oocyte retrieval was performed transvaginally 36 ± 2 h after the hCG injection. Fertilization was performed by conventional insemination or ICSI 4–6 h after oocyte retrieval based on the sperm quality. Up to two day 3 embryos or day 5–6 blastocysts were transferred by specified gynecologists following standardized procedures.

On the day of oocyte retrieval, luteal-phase support was initiated with oral dydrogesterone (40 mg/day) (Duphaston, Abbott, Chicago, IL, USA) or vaginal progesterone (90 mg/day) (Crinone, Merck Serono, Darmstadt, Germany) until the tenth week of pregnancy.

### 2.4. Biochemical Assessment

A 75 g oral glucose tolerance test (OGTT) and insulin-releasing test after an overnight fast of at least 8 h were conducted within six months before the initiation of COS. The fasting plasma glucose (FPG) and 2 h plasma glucose (2hPG) were quantified by the hexokinase method (Beckman Access Health Company, Chaska, MN, USA). The fasting plasma insulin (FPI) and 2 h plasma insulin (2hPI) were quantified by chemiluminescence using the Immulite 1000 system (DPC, USA). HbA1c was measured using ion-exchange high-performance liquid chromatography (HLC-723 G8, Tosoh, Tokyo, Japan). The laboratory technicians were blinded to other information about the study population.

### 2.5. Pregnancy and Neonatal Follow-Up

Pregnancy was preliminarily diagnosed by the positive serum hCG test 14 days after transplantation and further confirmed by the observation of fetal heart activity in the gestational sac by transvaginal ultrasound 3–4 weeks after transplantation. A live birth was defined as the delivery of a live fetus beyond 28 weeks of gestation. After delivery, well-trained follow-up nurses collected information regarding the pregnancy outcomes (i.e., clinical pregnancy and live birth) and birth outcomes (i.e., gestational age, delivery mode, neonatal sex, and neonatal birthweight) by telephone interviews.

### 2.6. Statistical Analysis

Statistical analyses were conducted with R software (version 4.0.3, R Foundation for Statistical Computing). The participants’ baseline reproductive and cycle characteristics were represented as the median (interquartile range (IQR)) or n (%). The homeostasis model assessment 2 estimate of insulin resistance (HOMA2-IR) was calculated with the HOMA2 Calculator (https://www.rdm.ox.ac.uk/about/our-clinical-facilities-and-mrc-units/DTU/software/homa, (accessed on 5 April 2023) version 2.2.4, University of Oxford, Oxford, UK) using FPG and FPI values [25]. The Gutt insulin sensitivity index was calculated as m/ (G × I), where m is a measure of glucose uptake during the OGTT calculated from body weight and fasting and 2 h glucose; G is the mean of fasting and 2-h glucose; and I is a log10 transformation of the mean of fasting and 2 h insulin [26]. The quantitative insulin sensitivity check index (QUICKI) was calculated as 1/[log (I_0_) + log (G_0_)], where I_0_ is the fasting insulin and G_0_ is the fasting glucose [27]. Differences in baseline reproductive and cycle characteristics between the singleton and twin groups were evaluated using the Kruskal–Wallis test for continuous variables and the Chi-squared test for categorical variables (or Fisher’s exact test where appropriate). The FPG, 2hPG, FPI, 2hPI, HbA1c, HOMA2-IR, Gutt index, and QUICKI were categorized into tertiles for the association analyses. When analyses required a normal distribution, FPG, 2hPG, FPI, 2hPI, and HbA1c were ln-transformed due to right skewness. 

Generalized linear models were established to investigate the associations between maternal preconception glucose metabolism indicators and neonatal birthweight in the singleton groups. Generalized estimate equations were established to explore the associations between maternal preconception glucose metabolism indicators and neonatal birthweight in the twin groups. Normal distribution and an identity link were applied for neonatal birthweight. The linear trend tests across tertiles were identified using an ordinal number (1, 2, and 3). The potential confounders were chosen if their associations with either birth outcomes or glucose metabolism were reported [28] or when the effect estimate led to a >10% change in the models to evaluate the associations between thyroid function indicators and neonatal birthweight [29]. Finally, we retained the following covariates in the models: preconception BMI and duration of infertility were examined as continuous variables; ovarian stimulation regimen (GnRH antagonist, long GnRHa, others), gestational age (<37 and 37–42 weeks), and delivery mode (vaginal delivery and caesarean section) were examined as dichotomous variables. All of the models were adjusted for the same set of covariates for consistency.

For better interpretation, the results of the total study population were also presented as marginal means using the R package “emmeans” (version 1.6.2-1). To explore the possible nonlinear effects, generalized additive models were established to evaluate the associations between maternal preconception glucose metabolism indicators and neonatal birthweight in the singleton and twin groups. We further stratified the study population by preconception BMI (<25 vs. ≥25 kg/m^2^) and delivery mode (vaginal delivery and caesarean section) to evaluate the possible modification effect. In addition, we conducted three sensitivity analyses to test the robustness of the results: (1) restricting the analyses to women with a BMI ≥18.5 kg/m^2^; (2) restricting the analyses to women applying stimulation cycles; (3) restricting the analyses to women with day 3 embryos transferred. Two-sided *p*-values of <0.05 were considered statistically significant.

## 3. Results

### 3.1. Baseline Reproductive and Clinical Characteristics of the Study Population

The baseline reproductive and clinical characteristics of 190 PCOS mothers with singletons and 79 PCOS mothers with twins are presented in Table 1. Among the PCOS women with singletons and twins, the median maternal age was 31 and 30 years old, respectively, and the median preconception BMI was 25.1 and 24.6 kg/m^2^, respectively. Most of the women were diagnosed with primary infertility (75.3% of the singleton group, 82.3% of the twin group); used the GnRH antagonist protocol (77.9% of the singleton group, 74.7% of the twin group); and underwent conventional IVF (77.4% of the singleton group, 73.4% of the twin group). Almost all of them underwent embryo transfer on day 3 (94.7% of the singleton group, 100.0% of the twin group) with two embryos (87.4% of the singleton group, 100.0% of the twin group). There were significant differences in the number of embryos transferred, gestational age, delivery mode, neonatal sex, and birthweight between the singleton and twin groups (all *p* < 0.01).

### 3.2. Glucose Metabolism Indicators and Neonatal Birthweight

The associations between maternal preconception glucose metabolism indicators and neonatal birthweight of the singleton and twin groups among PCOS women are shown in Table 2 and Table S1. We observed a negative correlation between maternal preconception FPG and neonatal birthweight in the singleton group (*p* for trend = 0.04). Compared with women in the first tertile of FPG, women in the third tertile had a decrease in singleton birthweight of −161.30 g (95% CI: −316.02 g, −6.52 g). We also observed a correlation between elevated maternal preconception HbA1c and decreasing neonatal birthweight in the singleton group (*p* for trend = 0.04). Compared with women in the first tertile of HbA1c, women in the third tertile had a decrease in singleton birthweight of −165.60 g (95% CI: −318.76 g, −12.40 g). However, we found no significant association between glucose metabolism indicators and twin neonatal birthweight.

**Figure 1:**
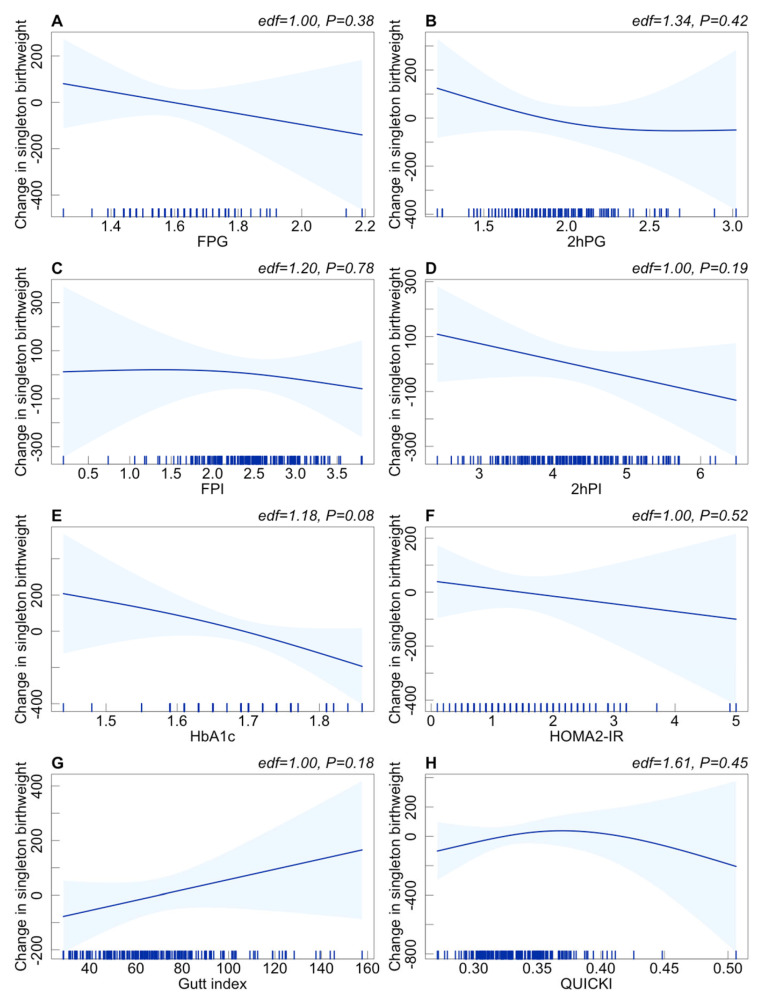
The potential nonlinear associations between glucose metabolism indicators and singleton birthweight among PCOS women undergoing their first IVF/ICSI cycles based on the generalized additive models using the “mgcv” R package (version R–4.0.3). (**A**–**H**): Change in singleton birthweight with the increase in FPG, 2hPG, FPI, 2hPI, HbA1c, HOMA2-IR, Gutt index, and QUICKI, respectively. Adjusted for preconception BMI (continuous), duration of infertility (continuous), ovarian stimulation regimen, gestational age, and delivery mode. The reference level (mazarine line) was set at the median. Blue shaded areas: 95% CI.

**Figure 2:**
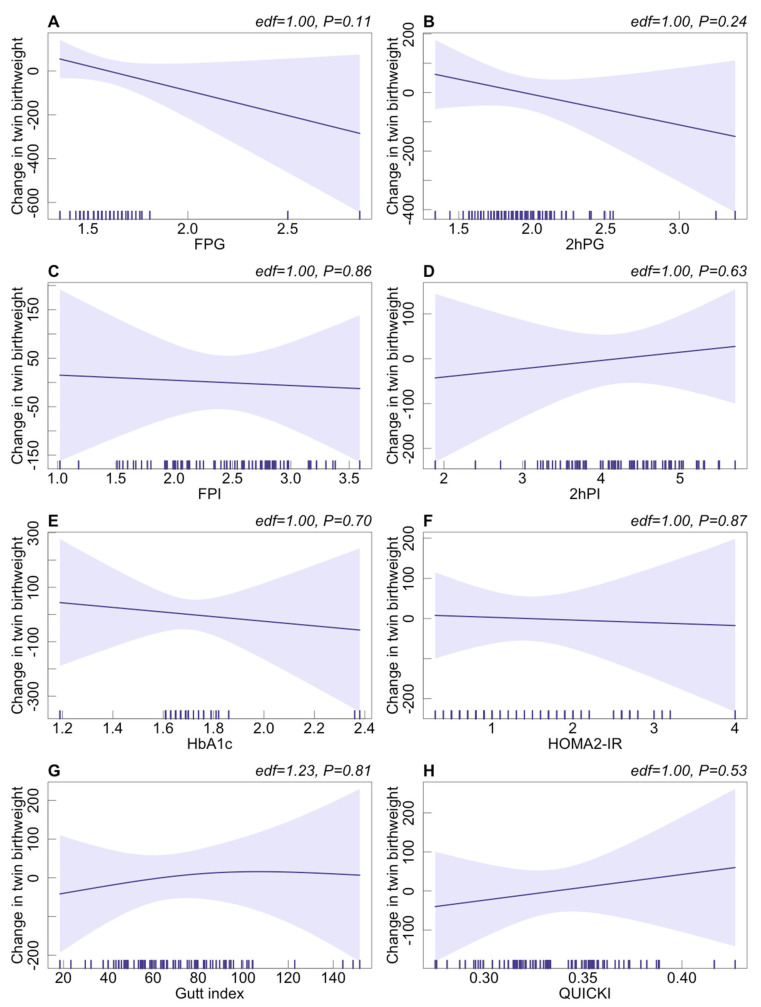
The potential nonlinear associations between glucose metabolism indicators and twin birthweight among PCOS women undergoing their first IVF/ICSI cycles based on the generalized additive models using the “mgcv” R package (version R–4.0.3). (**A**–**H**): Change in twin birthweight with the increase in FPG, 2hPG, FPI, 2hPI, HbA1c, HOMA2-IR, Gutt index, and QUICKI, respectively. Adjusted for preconception BMI (continuous), duration of infertility (continuous), ovarian stimulation regimen, gestational age, and delivery mode. The reference level (purple line) was set at the median. Lavender shaded areas: 95% CI.

We further assumed the potential nonlinear associations between maternal preconception glucose metabolism indicators and neonatal birthweight of the singleton and twin groups among PCOS women. As shown in Figure 1 and Figure 2, no significant nonlinear association between maternal preconception glucose metabolism indicators and neonatal birthweight was found in the singleton or twin group.

### 3.3. Stratified Analyses

**Figure 3:**
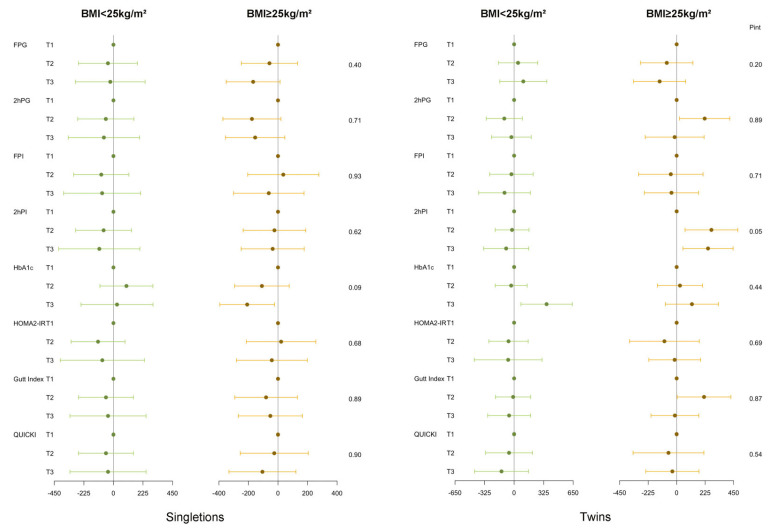
Regression coefficients (95% CI) for neonatal birthweight from glucose metabolism indicators among PCOS women undergoing their first IVF/ICSI cycles stratified by preconception BMI (<25 vs. ≥25 kg/m^2^). Adjusted for duration of infertility (continuous), ovarian stimulation regimen, gestational age, and delivery mode. Analyses of singletons and twins were based on the generalized linear models and generalized estimating equations, respectively.

Figure 3 shows the associations between maternal preconception glucose metabolism indicators and neonatal birthweight stratified by preconception BMI in PCOS women. We found an overweight-specific nonmonotonic association between elevated maternal preconception 2hPI and increased twin birthweight (*p* for interactions = 0.05). In overweight PCOS women, the estimated mean differences in twin neonatal birthweight when the second and third tertiles were compared with the first tertile of 2hPI were 274.30 g (95% CI: 66.18 g, 482.50 g; *p* < 0.01) and 247.60 g (95% CI: 49.65 g, 445.50 g; *p* = 0.01), respectively. 

**Figure 4:**
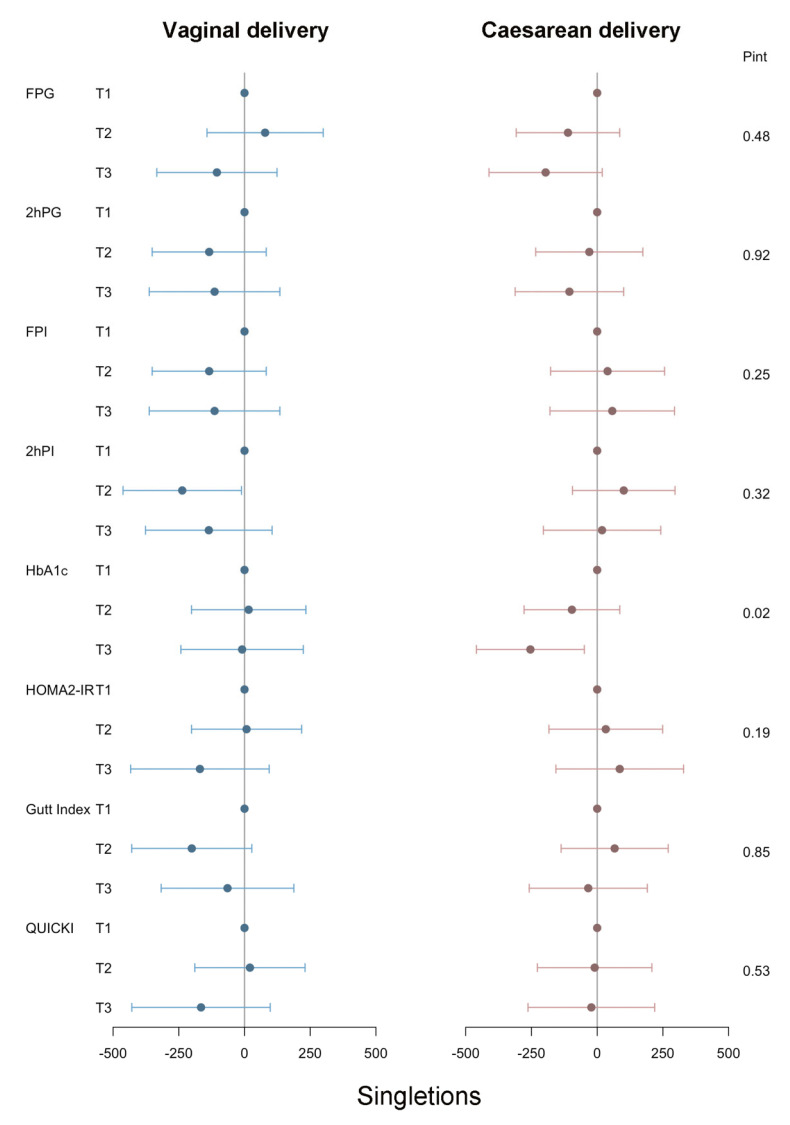
Regression coefficients (95% CI) for neonatal birthweight from glucose metabolism indicators among PCOS women undergoing their first IVF/ICSI cycles stratified by delivery mode (< vaginal delivery vs. ≥ caesarean delivery). Adjusted for preconception BMI (continuous), duration of infertility (continuous), ovarian stimulation regimen, and gestational age. Analyses of singletons and twins were based on the generalized linear models and generalized estimating equations, respectively.

Figure 4 shows the associations between maternal preconception glucose metabolism indicators and singleton neonatal birthweight stratified by delivery mode in PCOS women. We found a caesarean-specific association between elevated maternal preconception HbA1c and decreased singleton birthweight (*p* for interactions = 0.02). In PCOS women undergoing caesarean sections, the estimated mean differences in singleton neonatal birthweight when the extreme tertiles were compared were −253.99 g (95% CI: −459.10 g, −48.90 g; *p* = 0.02).

### 3.4. Sensitivity Analyses

Although some significant effects were attenuated, the aforementioned significant effect of maternal preconception FPG and HbA1c and singleton neonatal birthweight remained largely unchanged when the analysis was limited to women with a BMI ≥ 18.5 kg/m^2^ (Table S2), women applying stimulation cycles (Table S3), and women with day 3 embryos transferred (Table S4).

## 4. Discussions

We observed significant adverse effects of maternal preconception FPG and HbA1c on singleton birthweight in PCOS women undergoing their first IVF/ICSI outcomes. In the stratified analyses, we further found an overweight-specific nonmonotonic positive association between maternal preconception 2hPI and twin birthweight and a caesarean-specific negative association between maternal preconception HbA1c and singleton birthweight. 

Abnormal glucose metabolism diagnosed during pregnancy has been proven to be associated with pregnancy and neonatal complications. Previous studies have shown that gestational maternal hyperglycemia is associated with preterm delivery, induction of labor, macrosomia, LGA infants, shoulder dystocia, neonatal jaundice, and respiratory distress syndrome [9,30]. Besides, women with gestational diabetes mellitus (GDM) have elevated amniotic fluid (AF) volumes, elevated AF glucose concentrations, and altered AF metabolic profiles [31,32], which are associated with fetal growth and neonatal birthweight [32,33]. Gestational maternal IR during pregnancy has also been elucidated to increase the risk of preterm delivery, macrosomia, LGA infants, pregnancy-induced hypertension syndrome (PIH), and caesarean sections among non-GDM women [20,34]. Furthermore, gestational maternal HbA1c within the normal range has been illustrated as an independent risk factor for preterm birth, macrosomia, and LGA infants [35]. Several studies have suggested that maternal genetic variation may alter neonatal birthweight. A Brazilian study examined the association between maternal single-nucleotide polymorphisms (SNPs) variants related to glucose homeostasis and the offspring’s birthweight. They found that the mutation of GCK rs1799884 was associated with a decreased neonatal birthweight in the miscegenated Brazilian population [18]. Another British study elucidated that the presence of the A allele at position −30 of the GCK beta-cell-specific promoter, present in 30% of the population, was associated with increased offspring birthweight [36].

However, there is a lack of studies on the effect of abnormal preconception glucose homeostasis and IR on neonatal outcomes, especially on neonatal birthweight. A population-based retrospective cohort study among 6,447,339 Chinese women indicated that women with preconception DM or impaired fasting glucose had higher risks of adverse pregnancy and neonatal outcomes, including spontaneous abortion, preterm delivery, macrosomia, SGA, and perinatal infant death [4]. Another exploratory post hoc analysis of a randomized controlled trial from the LIFEstyle study group in the Netherlands found that preconception HOMA-IR, an acknowledged indicator of IR, is an important predictor of singleton birthweight among obese women [7]. In this study, we focused on PCOS women undergoing IVF/ICSI treatment and found significant associations between maternal preconception FPG and HbA1c and singleton birthweight. In addition, we observed an overweight-specific association between elevated maternal preconception 2hPI levels and increased twin birthweight and a caesarean-specific association between elevated maternal preconception HbA1c levels and decreased singleton birthweight. These results provided evidence of the impact of abnormal preconception glucose metabolism on neonatal outcomes and emphasized the importance of preconception blood glucose and insulin testing in PCOS women.

Glucose metabolism disorders in PCOS women have been widely illustrated [10]. Some research showed that PCOS could increase the risk of type 2 DM (T2DM). Specifically, the incidence of T2DM is four-fold higher in women with PCOS compared to those without PCOS in a cohort study based on the Australian Longitudinal Study on Women’s Health (ALSWH) database [37]. However, the causal evidence between T2DM and PCOS has not been proven via MR analysis [38]. Wang et al. suggested that the relationship between T2DM and PCOS might be mediated by other features (i.e., obesity) [11]. The causal links between glucose metabolism (i.e., fasting insulin, IR) and PCOS have been proven by several MR analyses [11]. The upregulated DNA methylation in the promoter region of the gene encoding peroxisome proliferator-activated receptor-gamma 1 (PPARGC1A) is a potential metabolic risk factor for IR [39]. Besides, the relationship between PCOS and abnormal glucose metabolism seems complementary. Hyperandrogenism, as a typical feature of PCOS, has causal links with T2DM [40]. In turn, hyperinsulinemia and IR in PCOS can exacerbate the effect of the luteinizing hormone on the androgen production of theca cells, thus leading to elevated circulating concentrations of total and free testosterone and more severe manifestations of PCOS [41,42]. Moreover, insulin may drive adipose testosterone generation by increasing aldoketoredutase type 3 (AKR1C3) activity in subcutaneous adipose tissue of PCOS women with IR [43]. Several studies found that the dysbiosis of Bacteroides vulgatus in the gut microbiota contributed to IR in PCOS women [44,45]. 

In our study, maternal preconception FPG and HbA1c were associated with decreased singleton birthweight. In PCOS women undergoing a caesarean section, the effect of HbA1c was more significant. Abnormal glucose metabolism was supposed to cause adverse singleton birthweight via abnormal oogenesis and embryogenesis. Evidence from animal studies has proved that abnormal glucose homeostasis, DM, and IR can impair oocyte quality and early embryo development. Some studies have elucidated that IR contributes to oxidative stress (OS) and impaired mitochondrial function in germinal vesicle (GV) and metaphase II (MII) oocytes, thus resulting in GV oocyte apoptosis, spindle abnormalities and the chromosomal misalignment of MII oocytes, fertilization disorder, increased embryo fragmentation, and the arrest of embryo development [46]. DM has been proven to induce abnormal ER distribution patterns [47], reduced oocyte zona pellucida thickness [48], mtDNA mutations [49], mitochondrial dysfunction [50], energy metabolism defects [51,52], meiosis abnormality [53], and epigenetic alteration [8,51] in oocytes. Mitochondrial dysfunction and energy metabolism dysregulation are also observed in the cumulus cells of diabetes mice [54]. The reduction of SIRT3 protein in the oocytes of DM mice can cause meiotic defects via the SIRT3-dependent GSK3β pathway [55]. These results show that the effect of abnormal glucose metabolism on oocyte quality may lead to abnormal embryo development and adverse pregnancy outcomes. Data from human studies which focused on PCOS women also suggested that abnormal glucose metabolism had adverse effects on oocyte and embryo development outcomes during IVF/ICSI, including poor ovarian response, decreased number of retrieved oocytes, impairment of oocyte maturation, and pregnancy outcomes [56,57,58]. We also found that maternal 2hSI was associated with increased twin birthweight in overweight PCOS women, suggesting that 2hSI may induce lipid accumulation in twin neonates through the interaction effect with BMI. The regulation of glucose and lipid metabolism are usually intertwined [59]. Zhu et al. elucidated the bidirectional causality among HDL cholesterol, triglycerides, and fasting insulin by MR analysis [60]. Besides, increased fatty acid esterification has been found on the fetal side of the placenta in twin pregnancies when compared with singleton pregnancies, indicating a more active lipid transfer to the fetal circulation in response to the greater energy demand of twins [61].

To our knowledge, this is the first study to investigate the effect of maternal preconception glucose metabolism on neonatal birthweight in PCOS women undergoing IVF/ICSI cycles. We found that maternal preconception abnormal glucose indicators can alter the neonatal birthweight and stressed the importance of preconception blood glucose and insulin monitoring and management in PCOS women. However, some limitations of our study should be mentioned. First, due to the retrospective nature of the study, the potential bias in data collection may lead to the inaccurate interpretation of analyses. However, given that the data in our study are objective measures with specific definitions, the bias may be partially eliminated. Well-designed prospective studies and related basic research are still needed to confirm our findings in the future. Second, we only studied the glucose metabolism indicators at a single preconception time. The gestational glucose metabolism condition may significantly influence pregnancy and neonatal outcomes [9]. Hence, further studies considering maternal glucose metabolism during preconception and all trimesters of pregnancy will provide more conclusive evidence. Third, we did not consider the possible adjunctive therapy recommended for hyperglycemic or IR women before and during pregnancy by doctors, including exercise, diet, and medication. This information was not well collected in the electronic medical records, which would result in underestimating the risks.

## 5. Conclusions

In conclusion, we found maternal preconception FPG and HbA1c were inversely associated with singleton birthweight. We also observed an overweight-specific positive association between maternal preconception 2hPI and twin birthweight and a caesarean-specific negative association between maternal preconception HbA1c and singleton birthweight. Although the results of our research need further confirmation via more well-designed prospective cohort studies and experimental studies, we suggested that preconception abnormal glucose metabolism may increase the risk of adverse neonatal outcomes in PCOS women. The monitoring and management of preconception glucose homeostasis and IR are essential methods of improving the neonatal outcomes of PCOS women.

## Figures and Tables

**Table 1:** Baseline reproductive and cycle characteristics of the PCOS women with singleton and twin births (median (IQR) or n (%)).

Characteristics		Singletons	Twins	P ^a^
		N = 190	N = 79	
Age, years		31 (28–33)	30 (28–32)	0.21
Preconceptional BMI, kg/m^2^		25.1 (22.1–28.5)	24.6 (22.3–27.8)	0.95
AFC, n		17 (13–22)	17 (13–24)	0.37
AMH, ng/mL		5.5 (3.6–7.8)	5.6 (3.6–9.3)	0.52
Duration of infertility, years		3 (2–5)	3 (2–5)	0.53
FPG, mmol/L		4.9 (4.6–5.2)	4.8 (4.5–5.1)	0.44
2hPG, mmol/L		6.7 (5.6–8)	6.6 (5.7–7.8)	0.84
FPI, uIU/ml		11.7 (8.1–17.2)	11.9 (7.6–16.4)	0.64
2hPI, uIU/ml		66.9 (41.8–113.8)	67.8 (39.1–123.7)	0.98
HbA1c, %		5.4 (5.2–5.7)	5.4 (5.3–5.6)	0.68
HOMA2-IR		1.3 (0.9–1.9)	1.3 (0.8–1.8)	0.74
Gutt index		65.9 (54.1–85.4)	66.7 (52.6, 81.9)	0.98
QUICKI		0.33 (0.31–0.35)	0.33 (0.31–0.35)	0.39
Infertility type	Primary	143 (75.3%)	65 (82.3%)	0.21
	Secondary	47 (24.7%)	14 (17.7%)	
Ovulation induction protocol	Stimulation cycle	182 (95.8%)	77 (97.5%)	0.73
	Minimal-stimulation cycle	8 (4.2%)	2 (2.5%)	
Ovarian stimulation regimen	GnRH antagonist	148 (77.9%)	59 (74.7%)	0.32
	Long GnRHa	41 (21.6%)	18 (22.8%)	
	Others ^b^	1 (0.5%)	2 (2.5%)	
Insemination technique	IVF	147 (77.4%)	58 (73.4%)	0.49
	ICSI	43 (22.6%)	21 (26.6%)	
Timing of embryo transfer	Day 3	180 (94.7%)	79 (100.0%)	0.11
	Day 5	7 (3.7%)	0 (0.0%)	
	Day 6	3 (1.6%)	0 (0.0%)	
Transferred embryo number, n	1	24 (12.6%)	0 (0.0%)	<0.01
	2	166 (87.4%)	79 (100.0%)	
Gestational age, weeks	<37	25 (13.2%)	43 (54.4%)	<0.001
	37–42	165 (86.8%)	36 (45.6%)	
Delivery mode	Vaginal delivery	90 (47.4%)	9 (11.4%)	<0.001
	Caesarean	100 (52.6%)	70 (88.6%)	
Neonatal sex	Male	98 (51.6%)	120 (75.9%)	<0.001
	Female	71 (48.4%)	59 (24.1%)	
Birthweight, g	<2500	13 (6.8%)	78 (49.4%)	<0.001
	2500–4000	167 (87.9%)	80 (50.6%)	
	≥4000	10 (5.3%)	0 (0.0%)	

Abbreviations: PCOS, polycystic ovary syndrome; IQR, interquartile range; BMI, body mass index; AFC, antral follicle count; AMH, anti-Müllerian hormone; FPG, fasting plasma glucose; 2hPG, 2 h plasma glucose; FPI, fasting plasma insulin; 2hPI, 2 h plasma insulin; HbA1c, glycosylated hemoglobin type A1C; HOMA2-IR, homeostasis model assessment 2 estimate of insulin resistance; QUICKI, quantitative insulin sensitivity check index; GnRH, gonadotropin-releasing hormone; GnRHa, gonadotropin-releasing hormone agonist; IVF, in vitro fertilization; ICSI, intracytoplasmic sperm injection. ^a^ p-values comparing the differences between the singleton group and twin group. ^b^ Other protocols include the short GnRHa protocol, ultrashort GnRH antagonist protocol, and clomiphene citrate protocol.

**Table 2:** Regression coefficients (95% CI) for neonatal birthweight by glucose metabolism indicators among PCOS women undergoing their first IVF/ICSI cycles ^a^.

Glucose Metabolism Indicators	β (95% CI)	
	Singletons ^b^	Twins ^c^
	N = 190	N = 79
**FPG**		
T1	Ref.	Ref.
T2	–60.30 (–204.38, 83.75)	–34.86 (–183.44, 113.70)
T3	–161.30 (–316.02, –6.52)	–48.13 (–193.74, 97.50)
*p* for trend	0.04	0.49
**2hPG**		
T1	Ref.	Ref.
T2	–105.10 (–252.17, 42.00)	113.31 (–27.58, 254.20)
T3	–139.80 (–297.65, 18.10)	–68.91 (–224.15, 86.30)
*p* for trend	0.08	0.44
**FPI**		
T1	Ref.	Ref.
T2	22.40 (–131.24, 176.00)	–13.46 (–177.19, 150.30)
T3	–70.40 (–242.50, 101.70)	–74.70 (–228.99, 79.60)
*p* for trend	0.42	0.35
**2hPI**		
T1	Ref.	Ref.
T2	–74.00 (–220.76, 72.80)	113.05 (–24.87, 251.00)
T3	–64.60 (–228.57, 99.40)	71.46 (–76.82, 219.70)
*p* for trend	0.41	0.30
**HbA1c**		
T1	Ref.	Ref.
T2	–50.90 (–191.54, 89.70)	2.10 (–129.80, 134.00)
T3	–165.60 (–318.76, –12.40)	181.39 (8.60, 354.20)
*p* for trend	0.04	0.07
**HOMA2-IR**		
T1	Ref.	Ref.
T2	2.39 (–150.00, 154.70)	–67.49 (–230.66, 95.70)
T3	–49.02 (–228.00, 130.10)	–22.90 (–177.09, 131.30)
*p* for trend	0.58	0.71
**Gutt Index**		
T3	Ref.	Ref.
T2	–89.99 (–241.56, 61.60)	126.38 (–15.69, 268.50)
T1	–89.46 (–257.52, 78.60)	–56.93 (–200.07, 86.20)
*p* for trend	0.28	0.58
**QUICKI**		
T3	Ref.	Ref.
T2	–2.73 (–154.94, 149.50)	–35.11 (–208.07, 137.80)
T1	–102.39 (–280.11, 75.30)	–69.03 (–221.26, 83.20)
*p* for trend	0.27	0.37

^a^ Adjusted for preconception BMI (continuous), duration of infertility (continuous), ovarian stimulation regimen, gestational age, and delivery mode. ^b^ Based on generalized linear models. ^c^ Based on generalized estimating equations

## Data Availability

The data analyzed in this study are available from the corresponding author upon reasonable request.

## Supplementary Materials

The following supporting information can be downloaded at: https://www.mdpi.com/article/10.3390/jcm12113863/s1, Table S1: Adjusted mean (95% CI) for neonatal birthweight by glucose metabolism indicators among PCOS women undergoing their first IVF/ICSI cycles; Table S2: Regression coefficients (95% CI) for neonatal birthweight by glucose metabolism indicators among PCOS women with preconception BMI ≥ 18.5 kg/m^2^ undergoing their first IVF/ICSI cycles; Table S3: Regression coefficients (95% CI) for neonatal birthweight by glucose metabolism indicators among PCOS women undergoing their first stimulated IVF/ICSI cycles; Table S4: Regression coefficients (95% CI) for neonatal birthweight by glucose metabolism indicators among PCOS women with Day 3 embryos tranferred undergoing their first IVF/ICSI cycles.

## References

[1] Danaei G., Finucane M.M., Lu Y., Singh G.M., Cowan M.J., Paciorek C.J., Lin J.K., Farzadfar F., Khang Y.-H., Stevens G.A. (2011). National, regional, and global trends in fasting plasma glucose and diabetes prevalence since 1980: Systematic analysis of health examination surveys and epidemiological studies with 370 country-years and 2.7 million participants. Lancet.

[2] Cho N.H., Shaw J.E., Karuranga S., Huang Y., da Rocha Fernandes J.D., Ohlrogge A.W., Malanda B. (2018). IDF Diabetes Atlas: Global estimates of diabetes prevalence for 2017 and projections for 2045. Diabetes Res. Clin. Pract..

[3] Metzger B.E., Lowe L.P., Dyer A.R., Trimble E.R., Chaovarindr U., Coustan D.R., Hadden D.R., McCance D.R., Hod M., McIntyre H.D. (2008). Hyperglycemia and adverse pregnancy outcomes. N. Engl. J. Med..

[4] Wei Y., Xu Q., Yang H., Yang Y., Wang L., Chen H., Anderson C., Liu X., Song G., Li Q. (2019). Preconception diabetes mellitus and adverse pregnancy outcomes in over 6.4 million women: A population-based cohort study in China. PLoS Med..

[5] Yajnik C.S., Deshmukh U.S. (2008). Maternal nutrition, intrauterine programming and consequential risks in the offspring. Rev. Endocr. Metab. Disord..

[6] Ajala O., Chik C. (2018). Ethnic differences in antepartum glucose values that predict postpartum dysglycemia and neonatal macrosomia. Diabetes Res. Clin. Pract..

[7] Wang Z., Nagy R.A., Groen H., Cantineau A.E., van Oers A.M., van Dammen L., Wekker V., Roseboom T.J., Mol B.W., Tietge U.J. (2021). Preconception insulin resistance and neonatal birth weight in women with obesity: Role of bile acids. Reprod. Biomed. Online.

[8] Bin Chen B., Du Y.-R., Zhu H., Sun M.-L., Wang C., Cheng Y., Pang H., Ding G., Gao J., Tan Y. (2022). Maternal inheritance of glucose intolerance via oocyte TET3 insufficiency. Nature.

[9] Ye W., Luo C., Huang J., Li C., Liu Z., Liu F. (2022). Gestational diabetes mellitus and adverse pregnancy outcomes: Systematic review and meta-analysis. BMJ.

[10] Joham A.E., Norman R.J., Stener-Victorin E., Legro R.S., Franks S., Moran L.J., Boyle J., Teede H.J. (2022). Polycystic ovary syndrome. Lancet Diabetes Endocrinol..

[11] Wang C., Wu W., Yang H., Ye Z., Zhao Y., Liu J., Mu L. (2022). Mendelian randomization analyses for PCOS: Evidence, opportunities, and challenges. Trends Genet..

[12] Bjercke S., Dale P.O., Tanbo T., Storeng R., Ertzeid G., Åbyholm T. (2002). Impact of insulin resistance on pregnancy complications and outcome in women with polycystic ovary syndrome. Gynecol. Obstet. Investig..

[13] Scholl T.O., Chen X., Gaughan C., Smith W.K. (2002). Influence of maternal glucose level on ethnic differences in birth weight and pregnancy outcome. Am. J. Epidemiol..

[14] Yang X., Zhang H., Dong L., Yu S., Guo Z., Hsu-Hage B.H.-H. (2004). The effect of glucose levels on fetal birth weight: A study of Chinese gravidas in Tianjin, China. J. Diabetes Complicat..

[15] Beksac M.S., Tanacan A., Hakli D.A., Ozyuncu O. (2018). Use of the 50-g glucose challenge test to predict excess delivery weight. Int. J. Gynecol. Obstet..

[16] Chivese T., Haynes M.C., van Zyl H., Kyriacos U., Levitt N.S., Norris S.A. (2021). The influence of maternal blood glucose during pregnancy on weight outcomes at birth and preschool age in offspring exposed to hyperglycemia first detected during pregnancy, in a South African cohort. PLoS ONE.

[17] Weedon M.N., Clark V.J., Qian Y., Ben-Shlomo Y., Timpson N., Ebrahim S., Lawlor D.A., Pembrey M.E., Ring S., Wilkin T.J. (2006). A common haplotype of the glucokinase gene alters fasting glucose and birth weight: Association in six studies and population-genetics analyses. Am. J. Hum. Genet..

[18] Alves L.N.R., Pereira M., dos Santos J.A., Santos E.D.V.W.D., de Carvalho G.Q., Santana J.D.M., Tavares E.A., Fernandes M.D.B., dos Santos D.B., Louro I.D. (2022). Investigation of maternal polymorphisms in genes related to glucose homeostasis and the influence on birth weight: A cohort study. J. Pediatr..

[19] Widness J.A., Schwartz H.C., Thompson D., King K.C., Kahn C.B., Oh W., Schwartz R. (1978). Glycohemoglobin (HbAIc): A predictor of birth weight in infants of diabetic mothers. J. Pediatr..

[20] Yamashita H., Yasuhi I., Fukuda M., Kugishima Y., Yamauchi Y., Kuzume A., Hashimoto T., Sugimi S., Umezaki Y., Suga S. (2014). The association between maternal insulin resistance in mid-pregnancy and neonatal birthweight in uncomplicated pregnancies. Endocr. J..

[21] Brown H.M., Green E.S., Tan T.C.Y., Gonzalez M.B., Rumbold A.R., Hull M.L., Norman R.J., Packer N.H., Robertson S.A., Thompson J.G. (2018). Periconception onset diabetes is associated with embryopathy and fetal growth retardation, reproductive tract hyperglycosylation and impaired immune adaptation to pregnancy. Sci. Rep..

[22] Rotterdam ESHRE/ASRM-Sponsored PCOS Consensus Workshop Group (2004). Revised 2003 consensus on diagnostic criteria and long-term health risks related to polycystic ovary syndrome. Fertil. Steril..

[23] Committee on Obstetric Practice American Institute of Ultrasound in Medicine Society for Maternal–Fetal Medicine (2017). Committee Opinion No 700: Methods for Estimating the Due Date. Obstet. Gynecol..

[24] Wang H., Gao H., Chi H., Zeng L., Xiao W., Wang Y., Li R., Liu P., Wang C., Tian Q. (2017). Effect of Levothyroxine on Miscarriage among Women with Normal Thyroid Function and Thyroid Autoimmunity Undergoing In Vitro Fertilization and Embryo Transfer: A Randomized Clinical Trial. JAMA.

[25] Wallace T.M., Levy J.C., Matthews D.R. (2004). Use and abuse of HOMA modeling. Diabetes Care.

[26] Gutt M., Davis C.L., Spitzer S.B., Llabre M.M., Kumar M., Czarnecki E.M., Schneiderman N., Skyler J.S., Marks J.B. (2000). Validation of the insulin sensitivity index (ISI (0, 120)): Comparison with other measures. Diabetes Res. Clin. Pract..

[27] Katz A., Nambi S.S., Mather K., Baron A.D., Follmann D.A., Sullivan G., Quon M.J. (2000). Quantitative insulin sensitivity check index: A simple, accurate method for assessing insulin sensitivity in humans. J. Clin. Endocrinol. Metab..

[28] Zong X., Wang H., Yang L., Guo Y., Zhao M., Magnussen C.G., Xi B. (2022). Maternal Pre-pregnancy Body Mass Index Categories and Infant Birth Outcomes: A Population-Based Study of 9 Million Mother–Infant Pairs. Front. Nutr..

[29] Greenland S. (1989). Modeling and variable selection in epidemiologic analysis. Am. J. Public Health.

[30] Farrar D., Simmonds M., Bryant M., Sheldon T.A., Tuffnell D., Golder S., Dunne F., Lawlor D.A. (2016). Hyperglycaemia and risk of adverse perinatal outcomes: Systematic review and meta-analysis. BMJ.

[31] Dashe J.S., Nathan L., McIntire D.D., Leveno K.J. (2000). Correlation between amniotic fluid glucose concentration and amniotic fluid volume in pregnancy complicated by diabetes. Am. J. Obstet. Gynecol..

[32] Zhou Y., Zhao R., Lyu Y., Shi H., Ye W., Tan Y., Li R., Xu Y. (2021). Serum and Amniotic Fluid Metabolic Profile Changes in Response to Gestational Diabetes Mellitus and the Association with Maternal–Fetal Outcomes. Nutrients.

[33] Xu Z.-M., Wu L.-F. (2006). Correlation between amniotic fluid glucose concentration and amniotic fluid volume and neonatal birth weight in pregnancy complicated by gestational diabetes mellitus. Zhonghua Fu Chan Ke Za Zhi.

[34] Tuuli M.G., Stout M.J., Macones G.A., Cahill A.G., Temming L.A. (2016). Maternal and Perinatal Outcomes in Women with Insulin Resistance. Am. J. Perinatol..

[35] Bi J., Ji C., Wu Y., Wu M., Liu Y., Song L., Khatiwada S.U., Yang S., Li B., Wang Y. (2020). Association Between Maternal Normal Range HbA1c Values and Adverse Birth Outcomes. J. Clin. Endocrinol. Metab..

[36] Weedon M.N., Frayling T.M., Shields B., Knight B., Turner T., Metcalf B.S., Voss L., Wilkin T.J., McCarthy A., Ben-Shlomo Y. (2005). Genetic regulation of birth weight and fasting glucose by a common polymorphism in the islet cell promoter of the glucokinase gene. Diabetes.

[37] Kakoly N.S., Earnest A., Teede H.J., Moran L.J., Joham A.E. (2019). The Impact of Obesity on the Incidence of Type 2 Diabetes Among Women with Polycystic Ovary Syndrome. Diabetes Care.

[38] Zhu T., Cui J., Goodarzi M.O. (2021). Polycystic Ovary Syndrome and Risk of Type 2 Diabetes, Coronary Heart Disease, and Stroke. Diabetes.

[39] Zhao H., Zhao Y., Ren Y., Li M., Li T., Li R., Yu Y., Qiao J. (2017). Epigenetic regulation of an adverse metabolic phenotype in polycystic ovary syndrome: The impact of the leukocyte methylation of PPARGC1A promoter. Fertil. Steril..

[40] Ruth K.S., Day F., Tyrrell J., Thompson D.J., Wood A.R., Mahajan A., Beaumont R.N., Wittemans L., Martin S., Busch A.S. (2020). Using human genetics to understand the disease impacts of testosterone in men and women. Nat. Med..

[41] Baillargeon J.-P., Carpentier A. (2007). Role of insulin in the hyperandrogenemia of lean women with polycystic ovary syndrome and normal insulin sensitivity. Fertil. Steril..

[42] Diamanti-Kandarakis E., Dunaif A. (2012). Insulin resistance and the polycystic ovary syndrome revisited: An update on mechanisms and implications. Endocr. Rev..

[43] O’Reilly M., Gathercole L., Capper F., Arlt W., Tomlinson J. (2015). Effect of insulin on AKR1C3 expression in female adipose tissue: In-vivo and in-vitro study of adipose androgen generation in polycystic ovary syndrome. Lancet.

[44] Qi X., Yun C., Sun L., Xia J., Wu Q., Wang Y., Wang L., Zhang Y., Liang X., Wang L. (2019). Gut microbiota–bile acid–interleukin-22 axis orchestrates polycystic ovary syndrome. Nat. Med..

[45] Yang Y.-L., Zhou W.-W., Wu S., Tang W.-L., Wang Z.-W., Zhou Z.-Y., Li Z.-W., Huang Q.-F., He Y., Zhou H.-W. (2021). Intestinal Flora is a Key Factor in Insulin Resistance and Contributes to the Development of Polycystic Ovary Syndrome. Endocrinology.

[46] Ou X.-H., Li S., Wang Z.-B., Li M., Quan S., Xing F., Guo L., Chao S.-B., Chen Z., Liang X.-W. (2012). Maternal insulin resistance causes oxidative stress and mitochondrial dysfunction in mouse oocytes. Hum. Reprod..

[47] Zhang C.-H., Qian W.-P., Qi S.-T., Ge Z.-J., Min L.-J., Zhu X.-L., Huang X., Liu J.-P., Ouyang Y.-C., Hou Y. (2013). Maternal diabetes causes abnormal dynamic changes of endoplasmic reticulum during mouse oocyte maturation and early embryo development. Reprod. Biol. Endocrinol..

[48] Li L., Jing Y., Dong M.-Z., Fan L.-H., Li Q.-N., Wang Z.-B., Hou Y., Schatten H., Zhang C.-L., Sun Q.-Y. (2020). Type 1 diabetes affects zona pellucida and genome methylation in oocytes and granulosa cells. Mol. Cell. Endocrinol..

[49] Li L., Wu C.-S., Hou G.-M., Dong M.-Z., Wang Z.-B., Hou Y., Schatten H., Zhang G.-R., Sun Q.-Y. (2018). Type 2 diabetes increases oocyte mtDNA mutations which are eliminated in the offspring by bottleneck effect. Reprod. Biol. Endocrinol..

[50] Jiang G., Zhang G., An T., He Z., Kang L., Yang X., Gu Y., Zhang D., Wang Y., Gao S. (2016). Effect of Type I Diabetes on the Proteome of Mouse Oocytes. Cell. Physiol. Biochem..

[51] Ma J.-Y., Li M., Ge Z.-J., Luo Y., Ou X.-H., Song S., Tian D., Yang J., Zhang B., Ou-Yang Y.-C. (2012). Whole transcriptome analysis of the effects of type i diabetes on mouse oocytes. PLoS ONE.

[52] Ratchford A.M., Chang A.S., Chi M.M.-Y., Sheridan R., Moley K.H. (2007). Maternal diabetes adversely affects AMP-activated protein kinase activity and cellular metabolism in murine oocytes. Am. J. Physiol. Endocrinol. Metab..

[53] Colton S.A., Pieper G.M., Downs S.M. (2002). Altered meiotic regulation in oocytes from diabetic mice. Biol. Reprod..

[54] Wang Q., Frolova A.I., Purcell S., Adastra K., Schoeller E., Chi M.M., Schedl T., Moley K.H. (2010). Mitochondrial dysfunction and apoptosis in cumulus cells of type i diabetic mice. PLoS ONE.

[55] Xin Y., Jin Y., Ge J., Huang Z., Han L., Li C., Wang D., Zhu S., Wang Q. (2021). Involvement of SIRT3-GSK3β deacetylation pathway in the effects of maternal diabetes on oocyte meiosis. Cell Prolif..

[56] Nafiye Y., Sevtap K., Muammer D., Emre O., Senol K., Leyla M. (2010). The effect of serum and intrafollicular insulin resistance parameters and homocysteine levels of nonobese, nonhyperandrogenemic polycystic ovary syndrome patients on in vitro fertilization outcome. Fertil. Steril..

[57] He Y., Lu Y., Zhu Q., Wang Y., Lindheim S.R., Qi J., Li X., Ding Y., Shi Y., Wei D. (2019). Influence of metabolic syndrome on female fertility and in vitro fertilization outcomes in PCOS women. Am. J. Obstet. Gynecol..

[58] Wang W., Tang X., Jiang Q., Niu Y., Wang Z., Wei D. (2022). Risk factors for clinical pregnancy loss after IVF in women with PCOS. Reprod. Biomed. Online.

[59] Chen L., Chen X.-W., Huang X., Song B.-L., Wang Y., Wang Y. (2019). Regulation of glucose and lipid metabolism in health and disease. Sci. China Life Sci..

[60] Zhu Z., Wang K., Hao X., Chen L., Liu Z., Wang C. (2022). Causal Graph Among Serum Lipids and Glycemic Traits: A Mendelian Randomization Study. Diabetes.

[61] Abascal-Saiz A., Fuente-Luelmo E., Haro M., de la Calle M., Ramos-Álvarez M.P., Perdomo G., Bartha J.L. (2021). Placental Compartmentalization of Lipid Metabolism: Implications for Singleton and Twin Pregnancies. Reprod. Sci..

