# Supplementary material for: Maternal Preconception Glucose Homeostasis and Insulin Resistance Are Associated with Singleton and Twin Birthweight of Neonates Conceived by PCOS Women Undergoing IVF/ICSI Cycles
Source: J Clin Med. 2023 Jun 5;12(11):3863. doi: 10.3390/jcm12113863 (PMC10254064; doi:10.3390/jcm12113863)
Supplement: Supplementary file 1 [file jcm-12-03863-s001.zip › jcm-2370218-supplementary.pdf]

Table S1. Adjusted mean (95% CI) for neonatal birthweight by glucose metabolism indicators among PCOS women undergoing their first IVF/ICSI cycles[a].

Glucose metabolism indicators	Mean birthweight (95% CI)	
	Singletons[b] N=190	Twins[c] N=79
**FPG**		
T1	2979 (2861, 3097)	2353 (2209, 2497)
T2	2919 (2795, 3043)	2318 (2154, 2482)
T3	2818 (2683, 2953)	2305 (2115, 2494)
P for trend	0.04	0.49
**2hPG**		
T1	2996 (2868, 3123)	2319 (2163, 2475)
T2	2891 (2775, 3006)	2432 (2288, 2576)
T3	2856 (2721, 2991)	2250 (2091, 2410)
P for trend	0.08	0.44
**FPI**		
T1	2929 (2800, 3058)	2360 (2195, 2525)
T2	2951 (2827, 3076)	2346 (2195, 2498)
T3	2859 (2726, 2991)	2285 (2119, 2451)
P for trend	0.42	0.35
**2hPI**		
T1	2960 (2834, 3085)	2271 (2110, 2432)
T2	2886 (2767, 3005)	2384 (2225, 2543)
T3	2895 (2757, 3033)	2342 (2177, 2507)
P for trend	0.41	0.30
**HbA1c**		
T1	2980 (2861, 3098)	2294 (2148, 2441)
T2	2929 (2806, 3052)	2297 (2151, 2442)
T3	2814 (2681, 2947)	2476 (2291, 2661)
P for trend	0.04	0.07
**HOMA2-IR**		
T1	2928 (2798, 3057)	2364 (2194, 2535)
T2	2930 (2807, 3053)	2297 (2144, 2449)
T3	2879 (2740, 3017)	2341 (2159, 2524)
P for trend	0.58	0.71
**Gutt Index**		
T3	2975 (2844, 3105)	2314 (2164, 2464)
T2	2885 (2767, 3003)	2441 (2287, 2594)
T1	2885 (2748, 3023)	2257 (2104, 2411)
P for trend	0.28	0.58
**QUICKI**		
T3	2947 (2820, 3073)	2363 (2197, 2530)
T2	2944 (2817, 3071)	2328 (2168, 2488)

T1	2844 (2706, 2982)	2294 (2122, 2467)
P for trend	0.27	0.37

[a] Adjusted for preconception BMI (continuous), duration of infertility (continuous), ovarian stimulation regimen, gestational age, and delivery mode.

[b] Based on generalized linear models.

[c] Based on generalized estimating equations.

Table S2. Regression coefficients (95% CI) for neonatal birthweight by glucose metabolism indicators among PCOS women with preconception BMI ≥18.5kg/m^2 undergoing their first IVF/ICSI cycles[a].

Glucose metabolism indicators	β (95% CI)<br>Singletons[b]<br>N=186	 Twins[c]<br>N=77
**FPG**		
T1	Ref.	Ref.
T2	−53.56 (−200.27, 93.15)	−31.15 (−180.92, 118.60)
T3	−155.59 (−310.47, −0.72)	−43.09 (−189.01, 102.80)
P for trend	0.05	0.54
**2hPG**		
T1	Ref.	Ref.
T2	−81.21 (−230.24, 67.80)	137.27 (−12.73, 287.30)
T3	−122.86 (−281.68, 36.00)	−53.41 (−209.98, 103.20)
P for trend	0.13	0.54
**FPI**		
T1	Ref.	Ref.
T2	45.30 (−110.90, 201.50)	−2.52 (−166.41, 161.40)
T3	−54.78 (−227.65, 118.10)	−69.72 (−224.55, 85.10)
P for trend	0.52	0.38
**2hPI**		
T1	Ref.	Ref.
T2	−65.65 (−213.32, 82.00)	136.26 (−5.59, 278.10)
T3	−56.09 (−220.13, 108.00)	88.43 (−60.86, 237.70)
P for trend	0.48	0.22
**HbA1c**		
T1	Ref.	Ref.
T2	−50.66 (−192.54, 91.22)	18.88 (−118.18, 155.90)
T3	−151.24 (−306.49, 4.01)	196.01 (21.68, 370.30)
P for trend	0.06	0.04
**HOMA2-IR**		
T1	Ref.	Ref.
T2	21.99 (−132.41, 176.40)	−58.77 (−222.17, 104.60)
T3	−37.19 (−216.52, 142.10)	−17.85 (−173.14, 137.40)
P for trend	0.69	0.77
**Gutt Index**		
T3	Ref.	Ref.
T2	−82.10 (−233.74, 69.50)	153.76 (6.11, 301.40)
T1	−81.97 (−249.65, 85.70)	−41.63 (−186.04, 102.80)
P for trend	0.33	0.71
**QUICKI**		
T3	Ref.	Ref.
T2	16.57 (−137.68, 170.80)	−28.33 (−200.97, 144.30)

T1	−92.14 (−270.10, 85.80)	−63.24 (−216.29, 89.80)
P for trend	0.33	0.42

[a] Adjusted for preconception BMI (continuous), duration of infertility (continuous), ovarian stimulation regimen, gestational age, and delivery mode.

[b] Based on generalized linear models.

[c] Based on generalized estimating equations.

Table S3. Regression coefficients (95% CI) for neonatal birthweight by glucose metabolism indicators among PCOS women undergoing their first stimulated IVF/ICSI cycles[a].

**Glucose metabolism indicators**	**β (95% CI)**<br>**Singletons**[b]<br>**N=182**	 **Twins**[c]<br>**N=77**
**FPG**		
T1	Ref.	Ref.
T2	−59.50 (−209.91, 90.90)	−37.24 (−186.53, 112.10)
T3	−141.60 (−303.14, 19.90)	−24.73 (−170.69, 121.20)
P for trend	0.09	0.69
**2hPG**		
T1	Ref.	Ref.
T2	−86.43 (−240.14, 67.30)	100.89 (−42.93, 244.70)
T3	−128.72 (−290.93, 33.50)	−92.04 (−246.69, 62.60)
P for trend	0.12	0.27
**FPI**		
T1	Ref.	Ref.
T2	34.73 (−123.96, 193.40)	−32.27 (−200.48, 135.90)
T3	−79.30 (−257.35, 98.80)	−103.46 (−256.46, 49.50)
P for trend	0.39	0.19
**2hPI**		
T1	Ref.	Ref.
T2	−64.23 (−216.68, 88.20)	90.87 (−47.03, 228.80)
T3	−80.66 (−249.29, 88.00)	53.70 (−97.11, 204.50)
P for trend	0.33	0.44
**HbA1c**		
T1	Ref.	Ref.
T2	−40.40 (−186.23, 105.40)	−24.46 (−159.81, 110.90)
T3	−168.94 (−326.56, −11.30)	149.21 (−29.42, 327.80)
P for trend	0.04	0.17
**HOMA2-IR**		
T1	Ref.	Ref.
T2	10.69 (−146.88, 168.30)	−89.98 (−256.83, 76.90)
T3	−47.73 (−230.45, 135.00)	−52.30 (−204.88, 100.30)
P for trend	0.62	0.45
**Gutt Index**		
T3	Ref.	Ref.
T2	−82.41 (−239.94, 75.10)	110.15 (−33.76, 254.10)
T1	−84.21 (−256.21, 87.80)	−79.04 (−222.05, 64.00)
P for trend	0.33	0.36
**QUICKI**		
T3	Ref.	Ref.
T2	3.60 (−153.51, 160.70)	−50.07 (−230.37, 130.20)

T1	−100.49 (−283.76, 82.80)	−100.28 (−251.07, 50.50)
P for trend	0.31	0.19

[a] Adjusted for preconception BMI (continuous), duration of infertility (continuous), ovarian stimulation regimen, gestational age, and delivery mode.

[b] Based on generalized linear models.

[c] Based on generalized estimating equations.

Table S4. Regression coefficients (95% CI) for neonatal birthweight by glucose metabolism indicators among PCOS women with Day 3 embryos tranferred undergoing their first IVF/ICSI cycles[a].

Glucose metabolism indicators	β (95% CI) Singletons[b] N=180	Twins[c] N=79
**FPG**		
T1	Ref.	Ref.
T2	−36.70 (−186.69, 113.28)	–34.86 (–183.44, 113.70)
T3	−175.65 (−338.16, −13.14)	–48.13 (–193.74, 97.50)
P for trend	0.04	0.49
**2hPG**		
T1	Ref.	Ref.
T2	−86.92 (−239.91, 66.07)	113.31 (–27.58, 254.20)
T3	−129.35 (−294.55, 35.85)	–68.91 (–224.15, 86.30)
P for trend	0.12	0.44
**FPI**		
T1	Ref.	Ref.
T2	42.77 (−117.95, 203.50)	–13.46 (–177.19, 150.30)
T3	−60.35 (−238.94, 118.25)	–74.70 (–228.99, 79.60)
P for trend	0.50	0.35
**2hPI**		
T1	Ref.	Ref.
T2	−54.68 (−208.58, 99.23)	113.05 (–24.87, 251.00)
T3	−51.87 (−220.91, 117.16)	71.46 (–76.82, 219.70)
P for trend	0.52	0.30
**HbA1c**		
T1	Ref.	Ref.
T2	−49.67 (−196.71, 97.38)	2.10 (–129.80, 134.00)
T3	−154.27 (−314.92, 6.39)	181.39 (8.60, 354.20)
P for trend	0.06	0.07
**HOMA2-IR**		
T1	Ref.	Ref.
T2	18.33 (−140.13, 176.79)	–67.49 (–230.66, 95.70)
T3	−40.25 (−225.25, 144.75)	–22.90 (–177.09, 131.30)
P for trend	0.69	0.71
**Gutt Index**		
T3	Ref.	Ref.
T2	−79.13 (−235.50, 77.24)	126.38 (–15.69, 268.50)
T1	−89.07 (−263.20, 85.06)	–56.93 (–200.07, 86.20)
P for trend	0.30	0.58
**QUICKI**		
T3	Ref.	Ref.
T2	10.68 (−147.24, 168.60)	–35.11 (–208.07, 137.80)

T1	−94.98 (−280.21, 90.26)	–69.03 (–221.26, 83.20)
P for trend	0.34	0.37

[a] Adjusted for preconception BMI (continuous), duration of infertility (continuous), ovarian stimulation regimen, gestational age, and delivery mode.

[b] Based on generalized linear models.

[c] Based on generalized estimating equations.
